# Scores of Health-Related Quality of Life Questionnaire Worsen Consistently in Patients of COPD: Estimating Disease Progression over 30 Years by SReFT with Individual Data Collected in SUMMIT Trial

**DOI:** 10.3390/jcm9082676

**Published:** 2020-08-18

**Authors:** Shinya Kawamatsu, Ryota Jin, Shogo Araki, Hideki Yoshioka, Hiromi Sato, Yasunori Sato, Akihiro Hisaka

**Affiliations:** 1 Clinical Pharmacology and Pharmacometrics, Graduate School of Pharmaceutical Sciences, Chiba University, 1-8-1, Inohana, Chuo-ku, Chiba 260-8675, Japan; s.kawamatsu.gifu@gmail.com (S.K.); ryota-j@chiba-u.jp (R.J.); shogo.1202.tandf@gmail.com (S.A.); ysokhdk@gmail.com (H.Y.); hiromi-s@chiba-u.jp (H.S.); 2 Clinical Operations, Japan Development, GlaxoSmithKline, Tokyo 107-0052, Japan; 3 Department of Preventive Medicine and Public Health, Keio University School of Medicine, Tokyo 160-8582, Japan; yasunori.sato@keio.jp

**Keywords:** chronic obstructive pulmonary disease, disease progression, health-related quality of life, smoking, statistical restoration of fragmented time-course

## Abstract

The aim of this study was to elucidate the lifelong disease progression of chronic obstructive pulmonary disease (COPD) with biomarker changes and identify their influencing factors, by utilizing a new analysis method, Statistical Restoration of Fragmented Time-course (SReFT). Individual patient data (*n* = 1025) participating in the Study to Understand Mortality and MorbidITy (SUMMIT, NCT01313676), which was collected within the observational period of 4 years, were analyzed. The SReFT analysis suggested that scores of St. George’s Respiratory Questionnaire and COPD assessment test, representative scores of the health-related quality of life (HRQOL) questionnaire, increased consistently for 30 years of disease progression, which was not detected by conventional analysis with a linear mixed effect model. It was estimated by the SReFT analysis that normalized forced expiratory volume in one second for age, sex, and body size (%FEV1) reduced for the initial 10 years from the onset of the disease but thereafter remained constant. The analysis of HRQOL scores and lung functions suggested that smoking cessation slowed COPD progression by approximately half and that exacerbation accelerated it considerably. In conclusion, this retrospective study utilizing SReFT elucidated the progression of COPD over 30 years and associated quantitative changes in the HRQOL scores and lung functions.

## 1. Introduction

Chronic obstructive pulmonary disease (COPD) is a chronic inflammatory lung syndrome that affects millions of people, and it is estimated to become the third leading cause of death worldwide by 2030 [1]. The primary etiology of COPD is smoking. Chronic inflammation induced by cigarette smoke damages the lungs, resulting in persistent airflow limitation, which is progressive and difficult to recover from [2]. Thus, influencing factors such as smoking status have a significant impact on the progression of the disease [3]. Patients with COPD routinely experience various unpleasant symptoms relating directly or indirectly to airflow limitation such as dyspnea, exercise limitation, cough, sputum, and psychoneurotic symptoms [4,5].

Key parameters of the airflow limitation in COPD are the forced expiratory volume in 1 s (FEV1) and the forced vital capacity (FVC). The values of FEV1/FVC ratio and FEV1 are used to diagnose and stage COPD. Health-related quality of life (HRQOL) questionnaires, such as St. George’s Respiratory Questionnaire (SGRQ) and COPD assessment test (CAT) [6,7], are also used for the assessment of COPD. The SGRQ was developed as a disease-specific assessment scale in COPD with a range of 0–100. CAT is a questionnaire that provides a semi-quantitative measure of a patient’s overall quality of life with a range from 0 to 40. In both questionnaires, higher scores denote a more severe impact of COPD on a patient’s life.

Patients with COPD generally demonstrate a decline in FEV1 over time, and frequencies of exacerbation and dyspnea have been often increased. Since 2011, the Global Initiative for Chronic Obstructive Lung Disease (GOLD) has proposed a more multidimensional approach considering the progression of COPD, and it recommends determining the treatment strategy with the HRQOL scores and exacerbation history in addition to FEV1 [8]. Several clinical studies have been conducted to analyze disease progression of COPD and its influencing factors [9,10]. Some studies have developed a longitudinal model with FEV1 [11,12], but FEV1 is known to be affected by height, sex and age [13]. It is thought that the use of %FEV1, which excludes these influencing factors, may simplify the model, but to the best of our knowledge, no model has been reported that explains lifelong changes in %FEV1 in patients with COPD. In addition, few studies have focused on decades-long changes in HRQOL, such as questionnaire scores, due to the practical barriers to performing a cohort study over several decades with questionnaire assessment [14].

In order to analyze long-term changes of important biomarkers in chronic diseases, we developed a unique analysis method, Statistical Restoration of Fragmented Time-course (SReFT), which restores long-term time courses from numerous short fragments by extensively extending an analysis method used in population pharmacokinetics. SReFT has been successfully applied for the analysis of Alzheimer’s disease [15]. Thus, SReFT allows us to develop a decades-long progression model for COPD without carrying out decades-long cohort studies.

The aim of this study was to develop a lifelong disease progression model with pulmonary function and HRQOL and identify its influencing factors by utilizing SReFT.

## 2. Materials and Methods

### 2.1. Data Collection

The study plan was reviewed and approved by the investigational ethical review board of the Graduate School of Pharmaceutical Sciences, Chiba University. Upon our request, individual participant-level trial data were provided from ClinicalStudyDataRequest.com (CSDR, https://clinicalstudydatarequest.com). We identified the Study to Understand Mortality and MorbidITy (SUMMIT, NCT01313676) using the following criteria:

- COPD clinical study, available in Jan-2017;
- Double-blind, placebo-controlled, randomized clinical trials;
- Enrolled COPD patients;
- Clinical trials lasting more than or equal to 48 weeks;
- Clinical trials with more than 3000 patients;
- Clinical trials with continuous pulmonary function and HRQOL data;
- Clinical trials with available blood test data.

Details of the SUMMIT trial including eligibility criteria have been reported elsewhere [16]. Data from patients inhaled with placebo were analyzed because the objective of this study was to analyze the natural progress of the disease without a particular treatment. In the SUMMIT trial, the use of COPD medications such as theophylline was allowed, although inhaled corticosteroids and inhaled long-acting bronchodilators were discontinued at least 48 h before trial entry. In this study, the patients were limited to those from European countries (*n* = 1003), Canada (*n* = 10) and Australia (*n* = 12) because HRQOL questionnaire was performed only in those countries. In the SUMMIT trial, current and ex-smokers were defined based on whether or not the patient quit smoking more than six months before the baseline measurement of SUMMIT trial. The exacerbation history was defined as whether patients experienced at least one exacerbation (with or without hospitalization) within one year prior to the screening visit (previous exacerbation), and it differs from the definition of exacerbator in the GOLD guideline of since 2014 [8]. In the SUMMIT trial, 6-min walk test was not performed.

### 2.2. Analysis by Linear Mixed-Effect Model

For comparison to the SReFT analysis, changes in biomarkers by observation period in the SUMMIT trial were analyzed by linear mixed-effect model (LMEM). The data were classified in accordance with the covariates adopted in the SReFT analysis. The LMEM analysis was performed using R 3.6.3.

### 2.3. Analysis by SReFT

The following double-exponential function is generally used in SReFT because of its flexibility to describe the evolution of biomarkers during disease progression [15]:

*f_i_* (*t*) = *α_i_ exp* (*β_i_ exp* (*γ_i_* × *d* × *τ*)) *exp* (*ε_i_*) (1)

*d* = *d_esm_* × *d_nex_* × *d_inter_* (2)

where *f_i_* (*t*) is the observed value of the *i* th biomarker at time *t* in the observation period, *τ* is the elapsed time since disease onset, {*α_i_*, *β_i_*, *γ_i_*} is the parameter set obeying a multivariate normal distribution with variance matrix ω^2^, *d* is a product of covariates that may affect the disease progression, and *ε_i_* is the residual error obeying normal distribution N(0, σ^2^). In the analysis, in addition to {*α_i_, β_i_, γ_i_*}, *τ* is estimated considering *t* for all subjects [15]. In this analysis, only the diagonal terms of ω^2^ were considered. Covariates *d_esm_*, *d_nex_*, and *d_inter_* are for ex-smoker, patients without previous exacerbation, and interaction between smoking status and exacerbation history, respectively.

In the present study, the biomarkers used for the SReFT analysis were the results of the pulmonary function test (%FEV1 and %FVC) and the scores of the questionnaire (SGRQ and CAT). The modified medical research council (mMRC) dyspnea scale is another well-known questionnaire for assessing conditions based on the patient reported outcome, but it was not used as the target biomarker of this analysis, because SReFT assumes continuous biomarker change but the evaluation of mMRC is only in 5 stages. The %FEV1 and %FVC were calculated considering age, height, and sex using the post-bronchodilator FEV1 and FVC, and normal predictive values, which were calculated using the NHANES III reference equation [13,17]. %FEV1 and %FVC were log-normalized, and SGRQ and CAT scores were normalized before the SReFT analysis. The measured value of each biomarker after 90 days from the start of the trial was used to ensure that any initial short-term increase did not affect analysis of disease progression. %FEV1 is known to decrease with disease progression, and 80% or smaller is accepted as moderate or greater airflow obstruction in the GOLD guideline. In the present study, this value was regarded as the onset of disease progression, designated as disease time 0. The SReFT analysis was performed using NONMEM 7.4.3. The algorithm for disease time calculation was implemented in a specialized PRED subroutine as “PRED_SReFT” (Supplemental Material). The consistency between calculations by NONMEM and Napp [15] was confirmed.

## 3. Results

**Figure 1:**
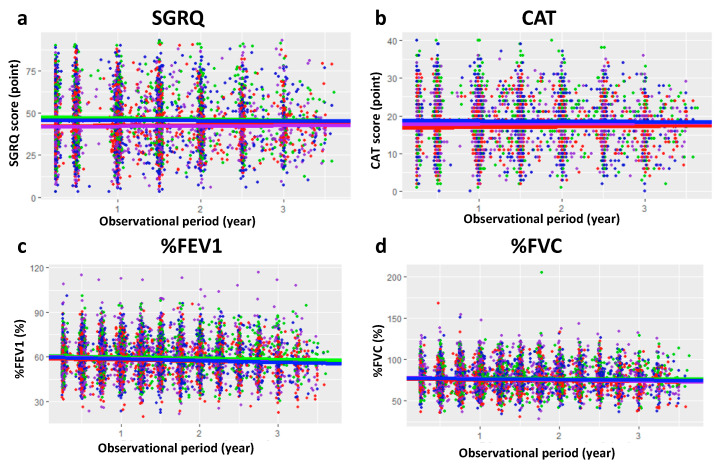
Changes of biomarkers within the observational period of SUMMIT trial. Observed data from SGRQ score (**a**), CAT score (**b**), %FEV1 (**c**), and %FVC (**d**) were plotted versus the original trial period. The evolutions of each sub-group’s biomarker estimated by using LMEM analysis were drawn. Blue: current smoker with previous exacerbation, Purple: current smoker without previous exacerbation, Green: ex-smoker with previous exacerbation, Red: ex-smoker without previous exacerbation. SUMMIT: Study to Understand Mortality and MorbidITy, SGRQ: St. George’s Respiratory Questionnaire, CAT: Chronic obstructive pulmonary disease assessment test, %FEV1: Percentage of predicted forced expiratory volume in 1 s, %FVC: Percentage of predicted forced vital capacity, LMEM: linear mixed-effect model.

In this study, we analyzed individual clinical data from 1025 patients who participated in the SUMMIT trial and dosed with placebo. Lung functions and questionnaire scores were used as biomarkers for the analysis. The evolution of each biomarker during the trial period and the result of LMEM analysis in the four groups divided by smoking status and exacerbation history are shown in Table 1 and Figure 1. In the analysis of %FEV1, ex-smokers had a slightly smaller slope than current smokers, and thus the progression of the disease might be slow in ex-smokers but to a very small degree. Changes in %FVC, SGRQ, and CAT varied between individuals; thus, it was difficult to capture consistent changes when the data were analyzed with the observational period in the SUMMIT trial.

Conversely, the SReFT analysis showed consistent increases in SGRQ and CAT with disease progression even in the base model without covariates. Because SReFT is an extension of nonlinear mixed-effect model analysis based on maximum likelihood estimation, covariates can be selected reasonably with the likelihood ratio theory using a χ^2^ test [18]. We selected the covariates that affect disease progression in stepwise forward-selection and backward-elimination methods. Smoking is well known to contribute to the development and progression of COPD [19]. In addition, patient exacerbation frequency, body mass index (BMI) and gender have also been accepted as factors affecting the progression of the disease [20,21,22]. As a result of stepwise forward-selection and backward-elimination of these candidate covariates, smoking status, exacerbation history, and the interaction between these two factors were adopted as significant covariates that affect COPD progression (Table 2). The race and the region in the world were not analyzed as covariates in this study since almost all subjects were from European countries.

**Figure 2:**
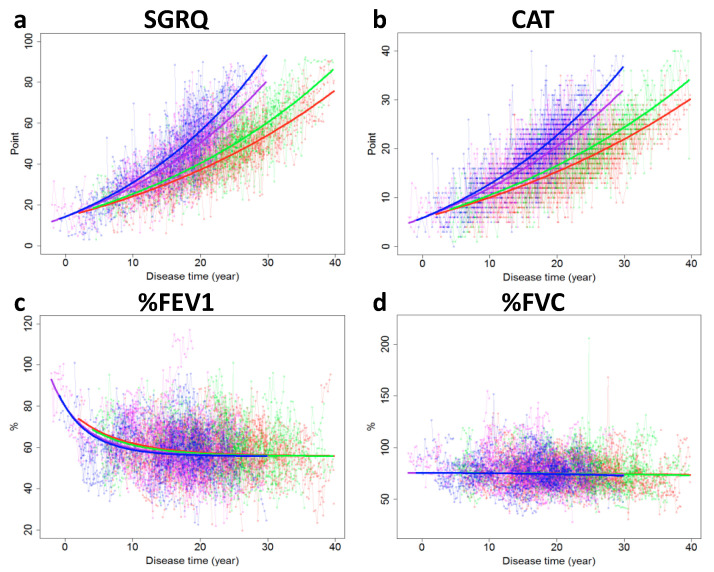
Restoration of fragmented changes in SGRQ score (**a**), CAT score (**b**), %FEV1 (**c**), and %FVC (**d**) by SReFT for long-term progression of COPD considering covariates of the patients. Blue: current smoker with previous exacerbation, Purple: current smoker without previous exacerbation, Green: ex-smoker with previous exacerbation, Red: ex-smoker without previous exacerbation. SGRQ: St. George’s Respiratory Questionnaire, CAT: Chronic obstructive pulmonary disease assessment test, %FEV1: Percentage of predicted forced expiratory volume in 1 s, %FVC: Percentage of predicted forced vital capacity, SReFT: Statistical Restoration of Fragmented Time-course, COPD: Chronic obstructive pulmonary disease.

The biomarker evolutions estimated using the final model are shown in Figure 2. The parameters estimated by SReFT are listed in Table 3. Over 30 years, changes in biomarkers in patients with COPD were successfully restored by using SReFT. It was estimated that the SGRQ and CAT scores increased consistently as the disease progressed, in contrast to the LMEM analysis. In addition, it was estimated that %FEV1 decreased slightly, soon after disease onset, and its decrease slowed down after 10–15 years, while %FVC did not change throughout the disease period.

Coefficients for ex-smokers and patients without previous exacerbation (*d_esm_* and *d_nex_*) were 0.711 and 0.906 (Table 3), respectively, which seems to suggest that the velocity of COPD progression was 0.711-fold less in ex-smokers than in current smokers. Similarly, the progression was 0.906-fold less in patients who did not experience exacerbation than in those who experienced exacerbation. SReFT estimates long-term trends based on the state of each subject at the time of observation; therefore, it should be noted that the results are based on the assumption that the patient’s backgrounds have not changed in the past and will not change in the future. Namely, SReFT cannot discriminate between smoking and nonsmoking periods in the past for ex-smokers. Actual disease progression in ex-smokers and patients with previous exacerbation will be discussed later. Although the interaction was regarded as statistically significant, its coefficient was almost 1.0, and it was nearly negligible.

To check the appropriateness of the SReFT analysis, we plotted the estimated values against the observed values and the deviations from the observed values against the estimated disease time (VPC plots). No obvious bias was found in these plots (Figure S1). Nevertheless, the validation of SReFT analysis is not simple because its validity depends on the selection of a hypothesis of the analysis as well as the data distributions in biomarkers and within disease time. To strengthen the validation, we generated random data that mimics the actual data based on the final model, fragmented, and then restored by SReFT. The results suggested that the disease time was successfully reconstituted from the fragmented virtual data (Figure S2).

To check for potential confounding factors in the analysis, the data were classified based on differences in adopted covariates (Table 4). There was no significant difference in patient characteristics, except for age, BMI, and smoking duration between subgroups (Table 4). Age and BMI were larger, and smoking duration was shorter in ex-smokers, but it was confirmed that these properties had no effect on disease progression when analyzed as an independent covariate. We also checked the bias in the distribution of the data when classified with adopted covariates. No significant bias between the subgroups was found in the analyzed data of each biomarker (Table S1).

Pulmonary function and questionnaire scores sometimes improve soon after the start of a clinical study of COPD, even though patients are assigned to placebo. In this analysis, data from 90 days after the start of the study were used to avoid such placebo effects. The original trial also used only the data after 90 days for the analysis of FEV1 [16]. To check the appropriateness of the 90 days period, several SReFT analyses were repeated by changing the deletion periods. The results were consistent when the data from the initial 90 days or more were deleted (Figure S3).

Since SReFT is theoretically an extension of population pharmacokinetic analysis, Bayesian inference can be applied to obtain full time biomarker trajectories for all individual patients [15]. Disease duration from the onset to the baseline measurement of the trial was estimated from the trajectories. Age at onset was estimated by subtracting the disease duration from the age at baseline. The disease duration was longer and age at onset was similar or slightly younger in ex-smokers than in current smokers (Table 4).

## 4. Discussion

COPD is characterized by persistent airflow limitation that is always progressive. Treatment is required for the remaining lifetime, so it is important to predict the future situation of the patient in particular conditions as precisely as possible and select the most probable remedy to delay progression. In the present study, we successfully estimated life-long disease progression from changes in the biomarkers collected in a clinical trial within a 4-year observation period and identified the factors affecting disease progression by applying SReFT analysis. We selected SGRQ and CAT scores as HRQOL status, and %FEV1 and %FVC as the pulmonary functions, for the target biomarkers of the analysis. The SReFT analysis suggested that the scores of SGRQ and CAT increased consistently over the entire period of the disease, which was not detected by conventional LMEM analysis. Quality of life decreases with disease severity [14] and with exacerbation [23]. Therefore, HRQOL scores such as SGRQ and CAT have been emphasized as important assessment tools for COPD in recent years [24]. Moreover, the GOLD guideline has recently included HRQOL scores as well as exacerbation and dyspnea histories for the diagnosis [8]. Nevertheless, FEV1 has been frequently used as the most reliable endpoint in clinical trials of COPD. This is because HRQOL scores usually did not change within approximately 1.5 years of typical observation period of clinical studies [25], and longer-term observations (e.g., decades-long) would be necessary to assess the changes. To the best of our knowledge, this is the first report to have clearly detected long-term changes in HRQOL scores in patients with COPD enrolled in a clinical study within a relatively restricted time period.

The present analysis suggested that ex-smokers showed a 0.711-fold slower disease progression overall than current smokers. In the patients analyzed in this study, the ex-smokers were 3–4 years older and their smoking years were 5–6 years shorter than that of current smokers (Table 4). In this clinical trial, patients with similar disease severity were enrolled, and it is probably the reason that ex-smokers tended to be older. Thus, 8–10 years in total may have passed since the ex-smokers ceased smoking, on average, if the current smokers and ex-smokers began smoking at a similar age. The average disease duration was estimated to be 21 years for ex-smokers (Table 4); therefore, they had continued smoking cessation most probably for approximately half of this period. A progression velocity change of 0.711-fold was caused by smoking cessation for only half of this period. Based on these considerations, it seems reasonable to conclude that COPD progression effectively slowed down by approximately half during smoking cessation. Previous studies have shown that smokers and ex-smokers differ in the rate of lung function decline by about 1.5- to 2-times [26] and are consistent with this study. Smoking causes not only deterioration of pulmonary functions but also in various respiratory system abnormalities [27], which may lead to a decrease in HRQOL. The present analysis is also proof of this possibility.

For patients with previous exacerbation, it is difficult to estimate the average period after exacerbation. The period after exacerbation should be considerably shorter than the whole disease time from onset because the recorded exacerbation was limited to those occurred within one year before screening visit (On the other hand, the period might be longer if the patient had experienced further exacerbations). For this reason, although the estimated *d_nex_*, 0.906, was near 1.0, the effect by exacerbation should not be underestimated. For ex-smokers, if the average period after the increase in exacerbation is 4 to 5 years, the progression of COPD is estimated to have accelerated double after the increase in exacerbation. The exacerbation history is now included for determination of the treatment strategy in the GOLD guideline in addition to FEV1 [8]. Therefore, the relationship between frequency of exacerbation and progression of COPD would need to be analyzed further if the data become available in the future.

Regarding the evolution of lung function, a trend of decrease in %FEV1 was observed within 10 years after the disease onset, that is, after %FEV1 dropped below 80%, but its decrease slowed down over this period. It has been reported that FEV1 in patients with mild COPD decreases faster than that in patients with severe COPD [28]; therefore, the results of this study support previous findings. No significant decrease was observed for %FVC throughout the entire disease period. %FVC is known to decrease in patients with severe COPD due to lung hyperinflation [29], but the SUMMIT trial did not enroll severe COPD patients, so the decrease may not have been observed. Although changes in lung function were not extensive in this analysis, caution should be taken where these changes may be affected by the inclusion and exclusion criteria of the trial. The HRQOL score consists of several symptom questions (e.g., breathlessness, limitation of movement, and cough), and lung function is thought to affect only a part of these items [30,31]. Thus, the reason why worsening of health status was observed, despite the pulmonary function being preserved, was that COPD affected many aspects of patient health status, more than just lung function.

The results of the present analysis might open potential applications in the future. The analysis suggested that the degree of biomarkers’ change/sensitivity would vary with stage of COPD. By applying this concept to clinical trials, it is worth considering planning “disease time-based” clinical trials. For example, in a study focusing on changes in pulmonary function, such as %FEV1, recruiting patients with early phase COPD by limiting their HRQOL score with the inclusion criteria would make it easier to assess changes in pulmonary function and may reduce the number of subjects. In addition, assessment and management of COPD that considers early changes in pulmonary functions and later ones in HRQOL scores may help to continue long-term treatment, keeping patients in better status. Furthermore, it may also be possible to evaluate therapeutic effects of a drug on the long-term progression of COPD as well as of other chronic diseases within a relatively short-term clinical trial using SReFT.

Our study has some limitations. SReFT is a new analysis method, and the outcomes should be interpreted with caution, although we tried to validate the analysis as much as possible. The present analysis was retrospective, and the SUMMIT trial was not designed for this analysis. All the results should be regarded as implicative and not proven yet. Some results may be biased due to restrictive enrollment criteria (e.g., SUMMIT trial enrolled only %FEV1 = 50–70% patients) and insufficient randomization. Prospective studies may be warranted to confirm the present results.

## 5. Conclusions

In the present study, we analyzed individual four years of observations of COPD patients using SReFT to estimate the long-term progression of the disease. The results of SReFT suggested that the HRQOL scores were consistently responsive to patients’ long-term disease progression and that smoking status and exacerbation history were strongly related to this progression. This study provided means of assessing long-term change in HRQOL scores for the first time within a limited period of clinical trials and supports the utility of the scores that have been reassessed in recent treatment guidelines. The findings will provide new insights into disease management and drug development for COPD.

## Figures and Tables

**Table 1:** Estimated parameters for the progression of COPD by LMEM.

Biomarker	Smoking Status	Previous Exacerbation *	Intercept	Slope
SGRQ	Ex-smk	Yes	42.1 (0.935)	0.355 (0.369)
	Ex-smk	No	47.5 (1.06)	−0.549 (0.468)
	Smk	Yes	42.1 (0.893)	0.213 (0.445)
	Smk	No	45.7 (1.24)	−0.127 (0.525)
CAT	Ex-smk	Yes	16.8 (0.374)	0.176 (0.150)
	Ex-smk	No	18.6 (0.483)	−0.0373 (0.216)
	Smk	Yes	17.8 (0.353)	0.0966 (0.176)
	Smk	No	18.8 (0.505)	−0.0995 (0.243)
%FEV1	Ex-smk	Yes	58.7 (0.612)	−0.710 (0.246)
	Ex-smk	No	60.6 (0.671)	−0.735 (0.331)
	Smk	Yes	60.5 (0.625)	−1.28 (0.272)
	Smk	No	60.0 (0.729)	−1.17 (0.353)
%FVC	Ex-smk	Yes	76.1 (0.827)	−0.710 (0.329)
	Ex-smk	No	77.0 (0.922)	−0.0832 (0.434)
	Smk	Yes	78.2 (0.849)	−1.27 (0.428)
	Smk	No	77.0 (0.923)	−0.651 (0.429)

Numbers in parentheses represent the standard error. COPD: Chronic obstructive pulmonary disease, LMEM: linear mixed-effect model, SGRQ: St. George’s Respiratory Questionnaire, CAT: Chronic obstructive pulmonary disease assessment test, %FEV1: Percentage of predicted forced expiratory volume in 1 s, %FVC: Percentage of predicted forced vital capacity, Ex-smk: Ex-smoker, Smk: Current smoker. * The patient experienced exacerbation within one year before baseline measurement of the trial.

**Table 2:** Process of covariate selection of SReFT analysis considering change in object function.

Model	Smoking Status	Exacerbation History	BMI at Baseline	Gender	Interaction	Object Function
Base model	N	N	N	N	NA	554.7
	Y	N	N	N	NA	413.2
	N	Y	N	N	NA	549.1
	N	N	Y	N	NA	544.9
	N	N	N	Y	NA	547.1
	Y	Y	N	N	N	403.8
Final model	Y	Y	N	N	Y	397.1
	Y	N	Y	N	N	419.2
	Y	N	Y	N	Y	399.8
	Y	N	N	Y	N	438.3
	Y	N	N	Y	Y	453.5
	Y	Y	Y	N	N	434.1
	Y	Y	Y	N	Y	414.3

“Y” denotes the factor incorporated in the model as a covariate. “N” denotes the factor was not incorporated in the model. “NA” denotes not available. SReFT: Statistical Restoration of Fragmented Time-course, BMI: Body mass index.

**Table 3:** Estimated parameters for the progression of COPD by SReFT.

Parameter		Biomarker			
		SGRQ	CAT	%FEV1	%FVC
*α*		−1.83	−1.80	1.62 *	0.0159
*β*		0.0835	0.0864	−0.347	−0.00131
*γ*		0.0411	0.0361	−0.19	0.0889
Inter-subject variability (ω)	*α*	0.142	0.151	0.00 **	0.829
	*β*	0.00 **	0.00 **	0.182	0.00 **
	*γ*	0.00464	0.00359	0.00 **	0.128
Intra-subject variability (σ)		0.529	0.584	0.515	0.524
Ex-smk	*d_esm_*	0.711			
Non-exa	*d_nex_*	0.906			
Interaction (Smk, Non-exa)	*d_inter_*	0.995			

COPD: Chronic obstructive pulmonary disease, SReFT: Statistical Restoration of Fragmented Time-course, SGRQ: St. George’s Respiratory Questionnaire, CAT: Chronic obstructive pulmonary disease assessment test, %FEV1: Percentage of predicted forced expiratory volume in 1 s, %FVC: Percentage of predicted forced vital capacity, Smk: Current smoker, Ex-smk: Ex-smoker, Non-exa: patients without previous exacerbation. * The alpha of %FEV1 was fixed to 1.62, which is the log-normalized value of 80% by definition of onset of the disease. ** The value was fixed to 0.00 to avoid excessive flexibility.

**Table 4:** Characteristics of patients in sub-group classified by adopted covariates.

Item	Current Smoker		Ex-Smoker		Statistic Difference
	Exacerbated *	Not Exacerbated	Exacerbated *	Not Exacerbated	
Number of Patients	221	296	236	272	N.S.
Number of Males <%>	161 <72.9>	208 <70.5>	172 <72.9>	200 <73.5>	N.S.
Age (At Baseline Measurement)	61.6 (7.6)	63.3 (7.8)	65.8 (8.2)	66.7 (6.5)	<0.001
Height (cm)	169 (9.5)	169 (8.9)	170 (8.8)	170 (8.7)	N.S.
BMI	28.3 (5.2)	27.7 (5.3)	29.4 (4.9)	30.0 (5.2)	<0.001
mMRC Score	2.25 (0.5)	2.19 (0.4)	2.28 (0.5)	2.21 (0.4)	N.S.
Baseline %FEV1 (%)	58.7 (6.6)	58.8 (7.8)	59.4 (7.7)	59.5 (7.2)	N.S.
Smoking Pack-Years	37.8 (20.8)	40.0 (20.2)	35.7 (23.1)	38.4 (22.3)	N.S.
Smoking Years	38.1 (10.4)	40.3 (10.2)	32.5 (11.4)	34.1 (10.8)	<0.001
Estimated Disease Duration **	14.6 (6.3)	15.3 (5.8)	21.3 (7.3)	21.4 (7.7)	<0.001
Estimated Age at COPD Onset	47.0 (9.9)	47.9 (10.0)	44.6 (10.9)	45.3 (9.7)	<0.001

Numbers in parentheses represent the standard deviation. The statistic comparison was performed with the Kruskal–Wallis test. N.S.: Not significant, BMI: Body mass index, mMRC: Modified Medical Research Council, COPD: Chronic obstructive pulmonary disease. * The patient experienced exacerbation within one year before screening visit of the trial. ** Period in years from estimated COPD onset to baseline measurement.

## Supplementary Materials

The following are available online at https://www.mdpi.com/2077-0383/9/8/2676/s1. Figure S1: Goodness-of-fit plot of the SReFT analysis with final model for visual predictive check. Figure S2: Appropriateness check of SReFT analysis by using virtual data, Figure S3: Check of initial placebo effect. Table S1: Summary of the properties of biomarker data classified by adopted covariates, Source code of PRED_SReFT.
