# Supplementary material for: Scores of Health-Related Quality of Life Questionnaire Worsen Consistently in Patients of COPD: Estimating Disease Progression over 30 Years by SReFT with Individual Data Collected in SUMMIT Trial
Source: J Clin Med. 2020 Aug 18;9(8):2676. doi: 10.3390/jcm9082676 (PMC7464378; doi:10.3390/jcm9082676)
Supplement: Supplementary file 1 [file jcm-09-02676-s001.pdf]

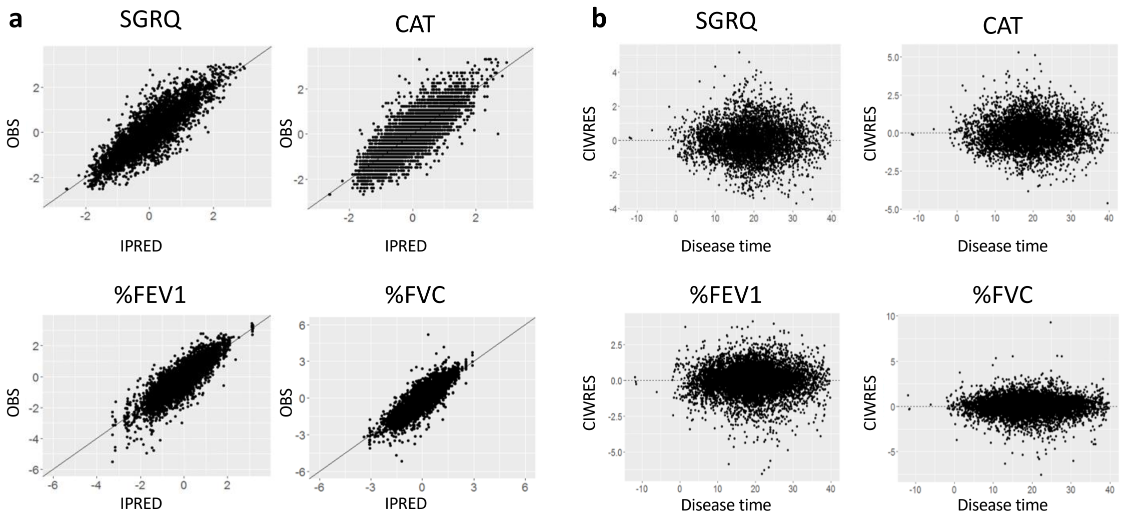

Figure S1. Goodness-of-fit plot of the SReFT analysis with final model for visual predictive check. Goodness-of-fit was checked with the plot of OBS vs. IPRED (a) and CIWRES vs. disease time (b). OBS, IPRED and CIWRES are shown as normalized value. SReFT: Statistical Restoration of Fragmented Time-course, OBS; Observed value, IPRED; Individual prediction, CIWRES; Weighted residuals for the individual patients, SGRQ: St. George's Respiratory Questionnaire, CAT: Chronic obstructive pulmonary disease assessment test, %FEV1: Percentage of predicted forced expiratory volume in 1 second, %FVC: Percentage of predicted forced vital capacity.

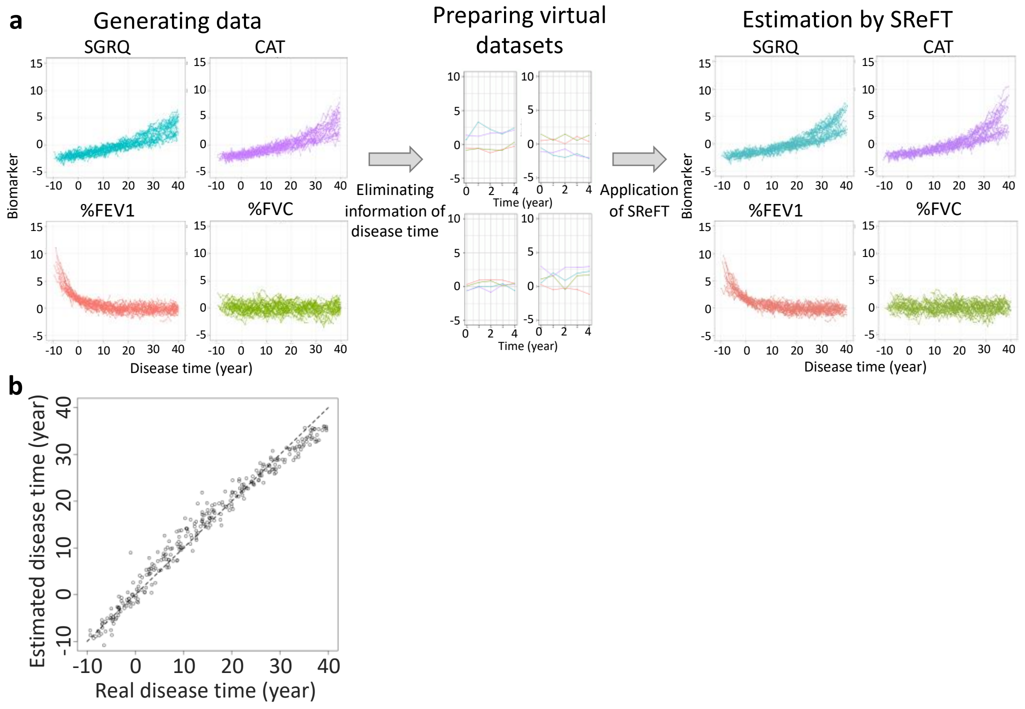

Figure S2. Appropriateness check of SReFT analysis by using virtual data. Virtual data of SGRQ, CAT, %FEV1 and %FVC were randomly generated adopting the disease times calculated by the final model of SReFT analysis for 300 patients (Left panel of a). All the data were fragmentated (Middle panel of a, only means were shown), and then reconstituted by SReFT (Right panel of a). Correlation of disease times before the fragmentation and reconstituted disease times is shown in panel b. SReFT: Statistical Restoration of Fragmented Time-course, SUMMIT: Study to Understand Mortality and MorbidITy, SGRQ: St. George's Respiratory Questionnaire, CAT: Chronic obstructive pulmonary disease assessment test, %FEV1: Percentage of predicted forced expiratory volume in 1 second, %FVC: Percentage of predicted forced vital capacity.

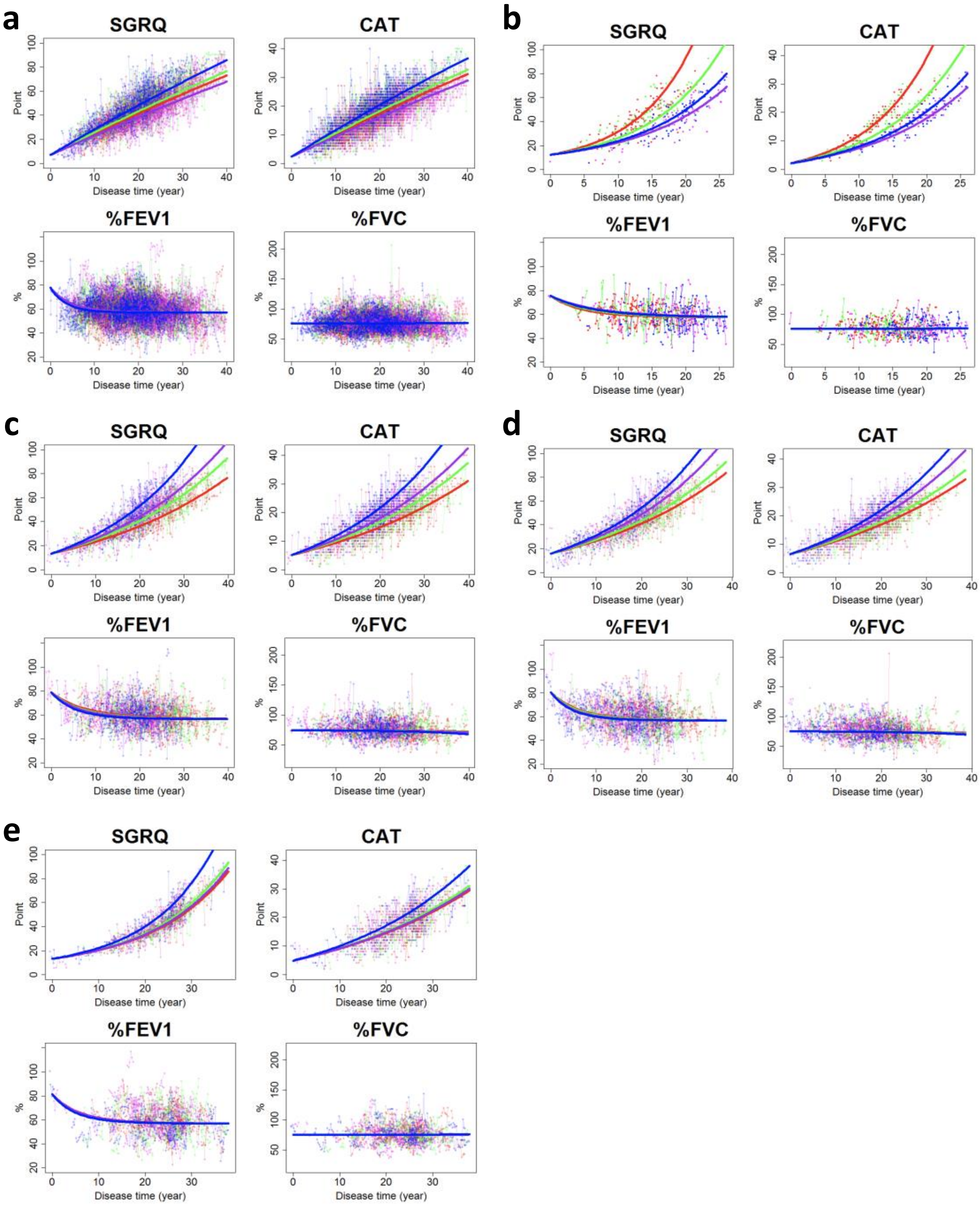

Figure S3. Check of initial placebo effect. The SReFT analyses were repeated changing restrictions of the observational period in the SUMMIT trial. (a) 0–1309 days (not restricted), (b) 0–90 days, (c) 90-360 days, (d) 360-720 days and (e) 720-1309 days. Blue: Current smoker with previous exacerbation, Purple: Current smoker without previous exacerbation, Green: Ex-smoker with previous exacerbation, Red: Ex-smoker without previous exacerbation. Please note that the progress of COPD was more rapid in the current smokers than in the ex-smokers in Panels a, c, d, and e. However, the order is different in Panel b, which suggests some perturbations by initial placebo effect. The analysis for 90-1309 days was adopted and presented in Figure 2. SReFT: Statistical Restoration of Fragmented Time-course, SUMMIT: Study to Understand Mortality and MorbidITy, COPD: Chronic obstructive pulmonary disease, SGRQ: St. George's Respiratory Questionnaire, CAT: Chronic obstructive pulmonary disease assessment test, %FEV1: Percentage of predicted forced expiratory volume in 1 second, %FVC: Percentage of predicted forced vital capacity.

Table S1. Summary of the properties of biomarker data classified by adopted covariates

Properties		Current smoker		Ex-smoker		statistic difference
		Exacerbated*	Not exacerbated	Exacerbated*	Not exacerbated	
SGRQ score	Number of observations	1081	1345	1145	1287	
	Observations per subject	4.9 (1.9)	4.5 (2.0)	4.9 (2.0)	4.7 (2.0)	N.S.
	Observed value	45.5 (17.0)	42.2 (13.8)	46.9 (14.1)	42.7 (13.9)	N.S.
CAT score	Number of observations	1083	1349	1148	1290	
	Observations per subject	4.9 (1.9)	4.6 (2.0)	4.9 (2.0)	4.7 (2.0)	N.S.
	Observed value	18.7 (6.6)	17.9 (5.4)	18.5 (5.8)	17.1 (5.3)	N.S.
%FEV1	Number of observations	1695	2114	1821	2017	
	Observations per subject	7.7 (3.5)	7.1 (3.7)	7.7 (3.7)	7.4 (3.8)	N.S.
	Observed value	58.5 (9.7)	59.0 (10.7)	59.8 (9.4)	57.8 (9.3)	N.S.
%FVC	Number of observations	1695	2115	1821	2017	
	Observations per subject	7.7 (3.5)	7.2 (3.7)	7.7 (3.7)	7.4 (3.8)	N.S.
	Observed value	76.3 (12.7)	76.8 (13.1)	76.9 (13.0)	75.4 (12.3)	N.S.

Numbers in parenthesis represent the standard deviation. The statistic comparison was performed with the Kruskal-Wallis test. SGRQ: St. George's Respiratory Questionnaire, CAT: Chronic obstructive pulmonary disease assessment test, %FEV1: Percentage of predicted forced expiratory volume in 1 second, %FVC: Percentage of predicted forced vital capacity, N.S.: Not significant

* The patient experienced exacerbation within one year before screening visit of the trial.

Source code of PRED_SReFT

```
module sreft
  use :: rocm_real, only: omega => varnf
  use :: nmprd_int, only: nthes_ => nwtht, netas_ => nweta, nepss_ => nweps
  use :: sizes, only: dpsize, isize
  use :: rocm_int, only: nindr => nindobs, indr1 => idxobsf, indr2 => idxobsl
  implicit none
  integer (kind=isize), save :: numbm, numcof, numsj
  integer (kind=isize), allocatable, save :: realid(:)
  real (kind=dpsize) :: plotmax = 40.0d0, plotmin = -15.0d0
  real (kind=dpsize) :: sum, wsum, ww, ma, mi, err, offsett, m, sigma
  real (kind=dpsize), allocatable, save :: offt(:)
contains

  subroutine initialize(id, meanx, meany, coun, a, b, c, bm, datrec, covt, covy)
    real (kind=dpsize) :: meanx(*), meany(*), coun(*), a(*), b(*), c(*), datrec(*), covt, covy(*)
    integer (kind=isize) :: id, i, bm

    sum = 0.0d0
    wsum = 0.0d0
    do i = 1, numbm
      if (coun(i)==0) cycle

      sigma = omega(netas_+i, netas_+i)
      ma = plotmax - meanx(i)
      mi = plotmin + meanx(i)
      if (b(i)==0.0d0) cycle
      if (bm==1) then
        if (c(i)==0.0d0) then
          m = (meany(i)-a(i))/(b(i)+covy(i))/covt
        else if ((meany(i)-a(i))/(b(i)/c(i)+covy(i))+1.0<0.0d0) then
          if (c(i)>0.0d0) then
            m = mi
          else
            m = ma
          end if
        else
          m = log((meany(i)-a(i))/(b(i)/c(i)+covy(i))+1.0)/c(i)/covt
        end if
      else
        if (c(i)==0.0d0) then
          m = (meany(i)-a(i)-covy(i))/b(i)/covt
        else if ((meany(i)-a(i)-covy(i))*c(i)/b(i)+1.0<0.0d0) then
          if (c(i)>0.0d0) then
            m = mi
          else
            m = ma
          end if
        else
          m = log((meany(i)-a(i)-covy(i))*c(i)/b(i)+1.0)/c(i)/covt
```

```
      end if
    end if
    if (m<mi) then
      m = mi
    else if (m>ma) then
      m = ma
    else
      m = m
    end if

    err = sigma/coun(i)
    if (c(i)/=0.0) then
      ww = err/(b(i)*covt*exp(c(i)*covt*m))**2
    else
      ww = err/(b(i)*covt)**2
    end if
    ww = 1/sqrt(ww)
    m = m - meanx(i)
    sum = sum + ww*m
    wsum = wsum + ww
  end do
  if (wsum>0.0d0) then
    offsett = sum/wsum
  else
    offsett = 0.0d0
  end if
  offt(id) = offsett
  realid(id) = datrec(1)
  return
end subroutine

subroutine eval(a, b, c, f, g, datrec, bm, id, meany, covt, covy)
  use :: prdims, only: gprd
  real (kind=dpsize) :: datrec(*), f, g(gprd, 1), a(*), b(*), c(*), time, meany(*), covt, covy(*)
  integer (kind=isize) :: bm, id, icall

  time = datrec(2) + offt(id)
  if (bm==1) then
    if (c(bm)==0) then
      f = a(bm) + (covy(bm)+b(bm))*covt*time
      g = 0.0d0
      g(bm, 1) = 1.0d0
      g(bm+numbm, 1) = covt*time
    else
      f = a(bm) + (b(bm)/c(bm)-covy(bm))*(exp(c(bm)*covt*time)-1.0d0)
      g = 0.0d0
      g(bm, 1) = 1.0d0
      g(bm+numbm, 1) = (exp(c(bm)*covt*time)-1.0d0)/c(bm)
      g(bm+2*numbm, 1) = (b(bm)/c(bm)-covy(bm))*covt*time*exp(c(bm)*covt*time) - &
        b(bm)/c(bm)**2*(exp(c(bm)*covt*time)-1.0d0)
    end if
```

```
    else
      if (c(bm)==0) then
        f = a(bm) + covy(bm) + b(bm)*covt*time
        g = 0.0d0
        g(bm, 1) = 1.0d0
        g(bm+numbm, 1) = covt*time
      else
        f = a(bm) + covy(bm) + b(bm)/c(bm)*(exp(c(bm)*covt*time)-1.0d0)
        g = 0.0d0
        g(bm, 1) = 1.0d0
        g(bm+numbm, 1) = (exp(c(bm)*covt*time)-1.0d0)/c(bm)
        g(bm+2*numbm, 1) = b(bm)/c(bm)*covt*time*exp(c(bm)*covt*time) - &
          b(bm)/c(bm)**2*(exp(c(bm)*covt*time)-1.0d0)
      end if
    end if
    return
  end subroutine

  subroutine makecsv
    implicit none
    integer (kind=isize) :: i

    open (13, file='offsetT.csv')
    write (13, '(a2, a2, a6, a2, a7)') 'id', ', ', 'realid', ',', 'offsetT'
    do i = 1, numsj
      write (13, '(i3, a2, i4, a2, e12.4)') i, ', ', realid(i), ',', offt(i)
    end do
    close (13)
    return
  end subroutine

end module

subroutine pred(icall, newind, theta, datrec, indxs, f, g, h)
  use :: sreft
  use :: prdims, only: gprd, hprd
  use :: nmprd_real, only: eta, eps
  use :: nmprd_int, only: newl2, iquit
  implicit none
  real (kind=dpsize) :: theta(*), datrec(*), f, g(gprd, 1), h(hprd, 1), cof(25), covt
  real (kind=dpsize), allocatable :: a(:), b(:), c(:), meanx(:), meany(:), coun(:), covy(:)
  integer (kind=isize) :: icall, newind, indxs(*), id, bm, smkbln, prevex

  if (icall==4) then
    if (newind/=2) then
      call simeta(eta)
      if (iquit==1) return
    end if
    if (newl2==1) then
      call simeps(eps)
      if (iquit==1) return
```

```
      end if
    else
      if (newind/=2) then
        call geteta(eta)
        if (iquit==1) return
        eps = 0.0d0
      end if
    end if

    if (newind==0) then
      numbm = nepss_
      numcof = (nthes_-nepss_*3)/numbm
      numsj = nindr
      if (icall==0) then
        allocate (offt(numsj), realid(numsj))
      end if
    end if
    allocate (a(numbm), b(numbm), c(numbm), meanx(numbm), meany(numbm), coun(numbm),
covy(numbm))
    id = datrec(1)
    bm = datrec(4)
    meanx = datrec(5:4+numbm)
    meany = datrec(5+numbm:4+2*numbm)
    coun = datrec(5+2*numbm:4+3*numbm)
    a = theta(1:numbm) + eta(1:numbm)
    b = theta(1+numbm:2*numbm) + eta(1+numbm:2*numbm)
    c = theta(1+2*numbm:3*numbm) + eta(1+2*numbm:3*numbm)
!define covariate
    if (numcof==0) then
      covt = 1.0d0
      covy = 0.0d0
    else
      smkbln = datrec(17)
            prevex = datrec(18)
      cof(1:25) = theta(13:37)
      if (smkbln==1) then
                        if (prevex==0) then
                    covt = (1.0d0 + cof(1))*(1.0d0 + cof(6))*(1.0d0 + cof(11))
                    covy = cof(2:5) + cof(7:10) + cof(14:17)
                        else
                    covt = (1.0d0 + cof(1))*(1.0d0 + cof(12))
                    covy = cof(2:5) + cof(18:21)
                        end if
      else
                        if (prevex==0) then
                    covt = (1.0d0 + cof(6))*(1.0d0 + cof(13))
                    covy = cof(7:10) + cof(22:25)
                        else
                    covt = (1.0d0 + 0.0d0)
                    covy = 0.0d0
                        end if
```

```
      end if
    end if
    if (newind<=1) call initialize(id, meanx, meany, coun, a, b, c, bm, datrec, covt, covy)
    call eval(a, b, c, f, g, datrec, bm, id, meany, covt, covy)
    f = f + eps(bm)
    h = 0.0d0
    h(bm, 1) = 1.0d0
    if (icall==3) call makecsv
    deallocate (a, b, c, meanx, meany, coun, covy)
    return
end subroutine
```
